# Head CT deep learning model is highly accurate for early infarct estimation

**DOI:** 10.1038/s41598-023-27496-5

**Published:** 2023-01-05

**Authors:** Romane Gauriau, Bernardo C. Bizzo, Donnella S. Comeau, James M. Hillis, Christopher P. Bridge, John K. Chin, Jayashri Pawar, Ali Pourvaziri, Ivana Sesic, Elshaimaa Sharaf, Jinjin Cao, Flavia T. C. Noro, Walter F. Wiggins, M. Travis Caton, Felipe Kitamura, Keith J. Dreyer, John F. Kalafut, Katherine P. Andriole, Stuart R. Pomerantz, Ramon G. Gonzalez, Michael H. Lev

**Affiliations:** 1 Data Science Office, Mass General Brigham, 100 Cambridge St, Suite 1303, Boston, MA 02114 USA; 2 Department of Radiology, Massachusetts General Hospital, Harvard Medical School, Boston, MA USA; 3 Diagnosticos da America SA (Dasa), Barueri, SP Brazil; 4 Department of Radiology, Brigham and Women’s Hospital, Harvard Medical School, Boston, MA USA; 5 Department of Neurology, Massachusetts General Hospital, Harvard Medical School, Boston, MA USA; 6 GE Healthcare, Chicago, IL USA

**Keywords:** Neuroscience, Neurology, Machine learning, Brain imaging

## Abstract

Non-contrast head CT (NCCT) is extremely insensitive for early (< 3–6 h) acute infarct identification. We developed a deep learning model that detects and delineates suspected early acute infarcts on NCCT, using diffusion MRI as ground truth (3566 NCCT/MRI training patient pairs). The model substantially outperformed 3 expert neuroradiologists on a test set of 150 CT scans of patients who were potential candidates for thrombectomy (60 stroke-negative, 90 stroke-positive middle cerebral artery territory only infarcts), with sensitivity 96% (specificity 72%) for the model versus 61–66% (specificity 90–92%) for the experts; model infarct volume estimates also strongly correlated with those of diffusion MRI (r^2^ > 0.98). When this 150 CT test set was expanded to include a total of 364 CT scans with a more heterogeneous distribution of infarct locations (94 stroke-negative, 270 stroke-positive mixed territory infarcts), model sensitivity was 97%, specificity 99%, for detection of infarcts larger than the 70 mL volume threshold used for patient selection in several major randomized controlled trials of thrombectomy treatment.

## Introduction

Stroke is a significant public health issue, affecting approximately 13.7 million people annually and the second major cause of death and disability worldwide^1^. Selection of stroke patients for treatment is typically based on both the: (i) clinical presentation and (ii) imaging findings, including but not limited to the presence or absence of intracranial hemorrhage (ICH), the presence of a target large vessel occlusion (LVO) in patients who are potential endovascular thrombectomy (EVT) candidates, and the size of the ischemic core. The management of acute ischemic stroke was revolutionized in 2018 with publication of the DAWN trial^2^. This study showed that the time window for safe and effective stroke treatment could be expanded from 6 to 24 h post symptom onset, with appropriate patient selection using “advanced” CT or MR imaging to detect and estimate the volume of irreversibly ischemic “core” infarction. Specifically, stroke patients with intracranial vascular occlusions and “small” (< 50 mL) estimated cores, treated with EVT, achieved a 49% rate of functional independence at 90-days, compared to only 13% with best medical therapy. A 50 mL infarct volume threshold was chosen as an enrollment criterion to minimize the risk of ICH as a treatment complication. The resulting effect size of 36% (49–13%) remains among the highest of any stroke trial to date, especially considering the treatment window of up to one-full day after symptom onset, with a “number-needed-to-treat” of only 2.8. In DAWN and related late-window (6-24 h) treatment studies, infarct volume was either estimated using maximally efficient, ground truth MR diffusion-weighted imaging (DWI) as the operational reference standard or approximated using CT perfusion imaging (CTP)^3–5^. Regardless of the imaging modality used for core estimation, however, all stroke clinical treatment trials have underscored the critical need for rapid, safe, highly sensitive and specific assessment, ideally minimizing cost, complexity, and technical variability^6,7^.

Only one major EVT clinical trial, MR CLEAN, which assessed treatment safety and efficacy in early stroke (< 6 h), used non-contrast CT (NCCT) exclusively to both rule out ICH prior to enrollment and to estimate infarct volume for subgroup analyses^7^. Unfortunately, both detection and volume estimation of early ischemic findings on NCCT—even by expert, subspecialty-certified neuroradiologists with decades of experience interpreting complex stroke scans—is significantly limited by the typically-subtle decreased X-ray attenuation and low contrast-to-noise ratios of acute infarcts. This poor conspicuity, attributable to the mildly reduced blood pool and early vasogenic edema of these developing lesions, is especially difficult to perceive in the first 3–6 h after stroke onset, before blood brain barrier breakdown becomes well established^8,9^. Even with interpretation by highly-trained readers using optimal image review display parameters, the sensitivity of NCCT for early (3–6 h) stroke detection has been reported to range as low as 43–71%, compared to 97% for DWI^8–10^. In a 2002 study comparing NCCT and DWI stroke detection within 3 h of symptom onset, sensitivity for expert readers was 61% by CT and 91% by DWI; for novice readers, sensitivity was 46% by CT and 81% by DWI, with CT described as “little better than flipping a coin”^11^. These results and others suggest that DWI is highly accurate for rapid, emergency department assessment of brain tissue viability; it identifies regions of reduced water diffusivity attributable to cytotoxic edema that are likely to be irreversibly infarcted even in the setting of early, robust restoration of critically ischemic cerebral blood flow^12^.

**Figure 1:**
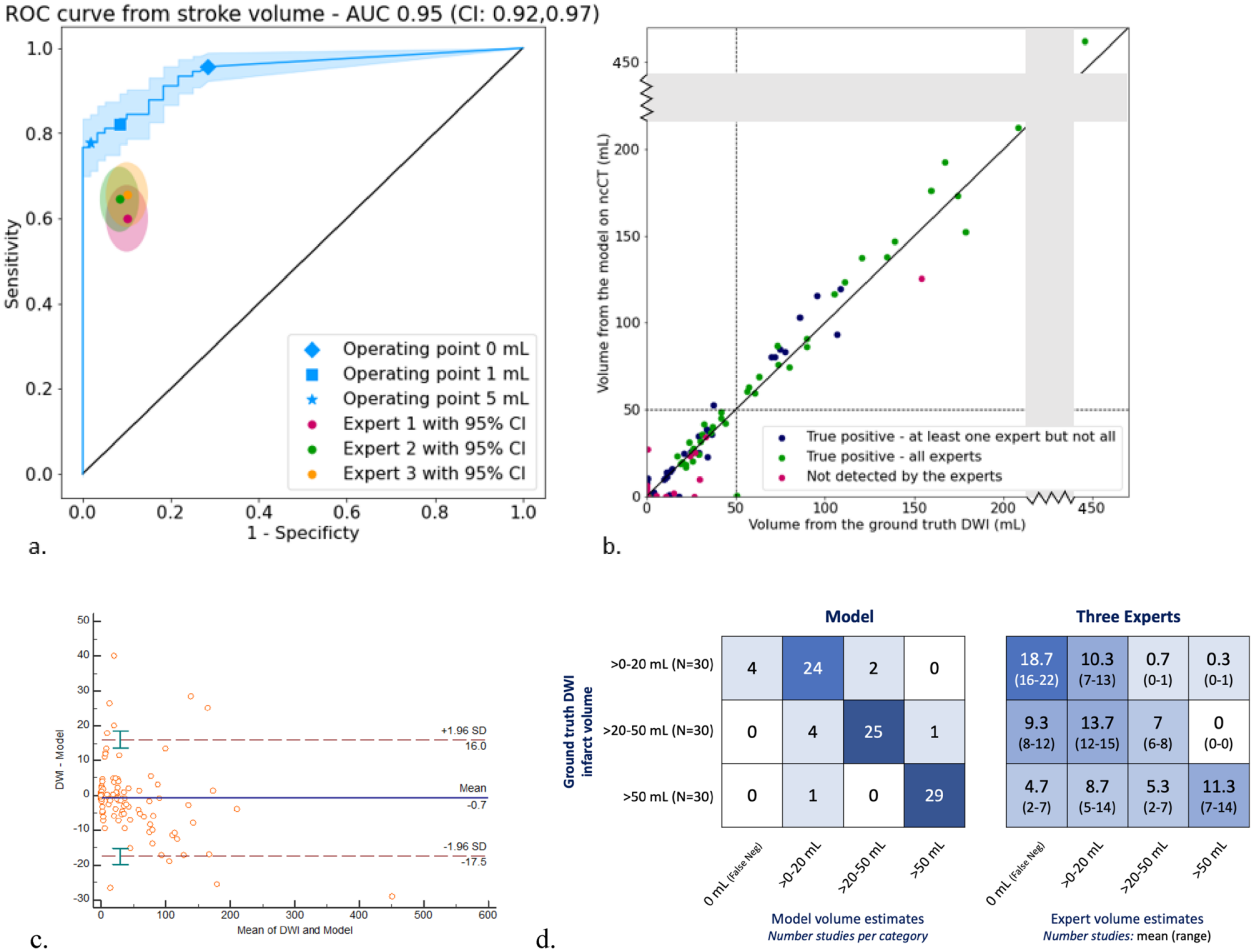
Model performance for infarct *detection* (**a**, ROC curve) and *delineation* (**b**, scatterplot; **c**, Bland–Altman plot; **d**, confusion matrices) in the “MCA-territory only” test set (see Table 1), based on DWI ground truth, compared to three human experts. (**a**) Model AUC was 0.95; sensitivity/specificity were 0.96/0.72 at a 0 mL-threshold operating point for infarct detection, 0.82/0.92 at a 1 mL-threshold, and 0.78/0.98 at a 5 mL-threshold for infarct detection, compared to mean reader sensitivity/specificity of 0.64/0.91. (**b**) Model infarct volume estimates strongly correlated with those of DWI ground truth (r^2^ > 0.98). As per the Bland–Altman plot (**c**), the model had excellent performance for estimating infarcts smaller versus larger than 50 mL (95%CI <  ± 17 mL), the volume threshold used for patient selection in major late window stroke treatment trials. Expert interrater Cohen’s kappa values ranged from 0.42 to 0.48, suggesting significant variability compared to the model, confirmed by the confusion matrices for volume segmentation (**d**, mean study-counts-per-category and ranges shown for the 3-experts; calculated at the model’s 0 mL-threshold for infarct detection).

**Figure 2:**
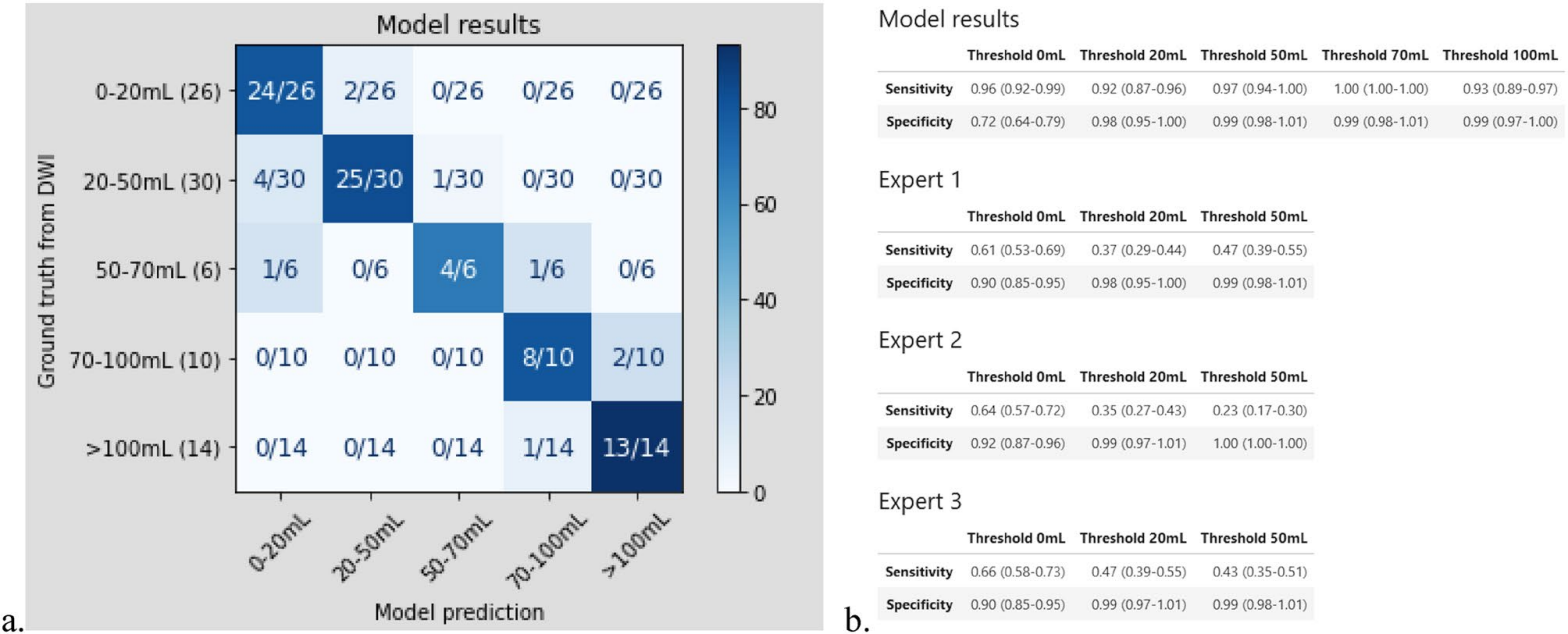
Detailed confusion matrix for additional volume stratification (as per Fig. 1d), at the 50–70 mL, 70–100 mL, and > 100 mL ranges, in the “MCA-territory only” test set (**a**, calculated at the model’s 0 mL-threshold for infarct detection). (**b**) Comparison of model sensitivity, specificity at the 20 mL, 50 mL, 70 mL, and 100 mL thresholds, versus the three individual expert raters at the 20 mL and 50 mL thresholds.

**Figure 3:**
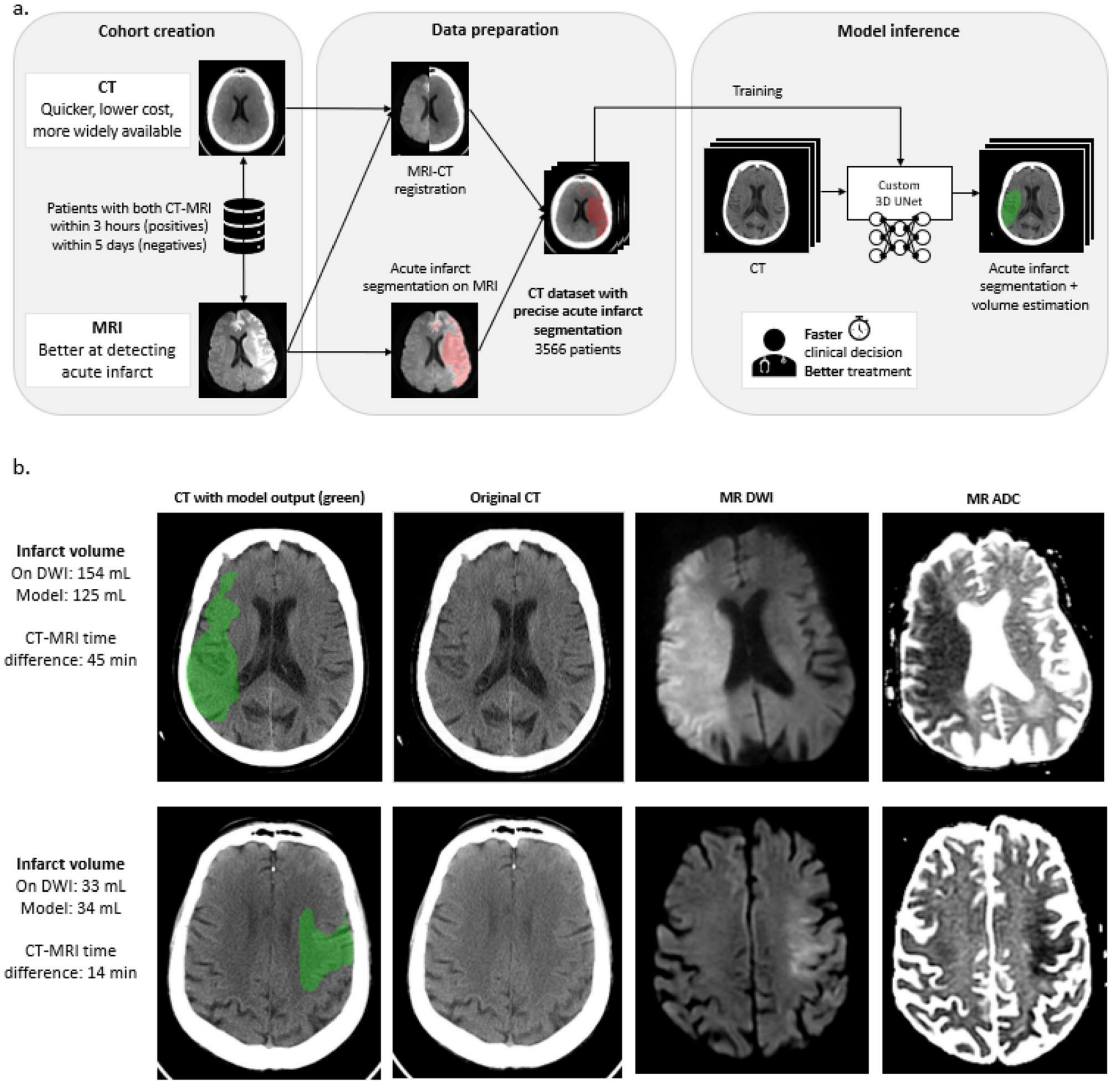
(**a**) Schematic representation of model development steps from cohort creation and data preparation to model inference training. (**b**) Examples of infarcts not detected by each of the three expert neuroradiologists, but accurately detected and delineated by the model.

In this study, we developed a deep learning model that detects, delineates, and estimates the volume of early acute infarction on NCCT, using diffusion MRI as ground truth (3,566 NCCT/MRI training patient pairs). We evaluated the performance of this model in two NCCT test sets (Table 1), the first a subset that included both stroke-negative and stroke-positive middle cerebral artery (MCA) territory only infarcts (n = 150, Figs. 1, 2 and 3) and the second an expanded set that included additional CT scans with more varied infarct locations (n = 364, Table 3). For the “MCA-territory-only” test set, we compared model performance with that of three expert neuroradiologists (mean 25-years’ experience, blinded to all other clinical/imaging data); expert review was randomized with a different order of presentation for each radiologist. The experts recorded the presence or absence of acute infarct and categorized estimated infarct sizes as > 0–20 mL,  > 20–50 mL, or  > 50 mL, using the formula [length x width x height]/2 (each in cm) to approximate volume in mL^13^.

**Table 1:** Dataset description with patient demographics and acquisition details. (Legend: no. = number, Std = standard deviation, M/F = male/female, IQR = inter-quartile range, mAs = milliampere-seconds, kVp = kilovoltage peak, MCA = middle cerebral artery, BG = basal ganglia, PCA = posterior cerebral artery, CorRad WM = corona radiata white matter, and ACA = anterior cerebral artery, LVO = large vessel occlusion [ICA, M1, M2]).

Patient demographics	Training	Validation	Full Test Set: Various Location Infarcts	Subset Test Set: MCA-territory only Infarcts
No. patients (stroke positive / negative)	3566 (1896 / 1670)	133 (66 / 67)	364 (270 / 94)	150 (90 / 60)
No. NCCT series	9528	338	364	150
Mean age (Std)	65 (17)	64 (17)	68 (15)	67 (17)
Gender: M / F	1779 (49.9%) / 1787 (50.1%)	74 (55.6%) / 59 (44.4%)	208 (57.1%) / 156 (42.9%)	73 (48.7%) / 77(51.3%)
Infarct volume ≥ 50 mL (no.)	334	17	43	30
Infarct volume ≥ 20 mL (no.)	641	19	88	60
Median DWI infarct volume, mL	9.4	5.2	8.8	29.6
Stroke laterality (no., %)			Left: 137/270 (51) Right: 128/270 (47) Unilateral: 265/270 (98) Bilateral: 5/270 (2)	Left: 46/90 (51) Right: 44/90 (49) Unilateral: 90/90 (100) Bilateral: 0/90 (0)
Stroke territory (no., %)			MCA: 183/270 (68) BG: 30/270 (11) PCA: 19/270 (7) CorRad WM: 14/270 (5) Brainstem: 13/270 (5) Cerebellum: 9/270 (3) ACA: 2/270 (1)	MCA: 90/90 (100)
LVO (no., %)				No LVO (11/90 = 12%) Bilateral LVO (2/90 = 2%) Left LVO (34/90 = 38%) *3 partial, 2 M3* Right LVO (29/90 = 32%) *1 partial, 1 M3* No CTA (14/90 = 16%)
**Acquisition**				
Mean time from NCCT-to-DWI: stroke positive / stroke negative	50 min / 16 h	50 min / 9 h	44 min / 16 h	35 min / 19 h
No. CT scans per vendor: GE Healthcare / Siemens (No. different CT models per vendor)	2509 / 1184	88 / 54	284 / 80	110 / 40 (5 / 3)
Range of years from which NCCT/DWI scans obtained	2001–2019	2002–2019	2002–2019	2002–2019
Mean X-ray scanning current	225 mA	217 mA	220 mA	220 mA
Mean X-ray scanning voltage	119 kVp	120 kVp	120 kVp	122 kVp

Our model, adapted from the U-Net architecture, takes an NCCT series as input and generates a segmentation mask of the early infarct changes, which is used to estimate infarct volume [23, *Methods*]. Model training relied on a large dataset of paired admission NCCT followed by ground truth DWI scans, acquired within a short time interval of one another. Infarcts were segmented semi-automatically, and segmentation masks for each pair were registered to the corresponding NCCT images (Fig. 3a).

## Results

The “MCA-territory only” test set included a subset of 150 scans from two different vendors and 8 different scanner models (Table 1, *Methods*). For this test set, our model significantly outperformed the three expert neuroradiologists for core detection of 150 NCCT scans (sensitivity 96%, specificity 72% model versus 61–66%, 90–92% experts, Figs. 1a and 2b). Of these 150 scans, 90 were stroke-positive and 60 stroke-negative; for the stroke-positive scans, median time (a) from symptom-onset-to-NCCT was 3.7 h (IQR 1.3–5.1 h; 14 time-points unavailable) and (b) from NCCT-to-DWI was 28 min (IQR 22–36 min); median time from NCCT-to-DWI for stroke-negative scans was 5.9 h (IQR 1.9–27.1).

Our model also approached the accuracy of ground truth DWI for core volume estimation (r^2^ > 0.98, Fig. 1b). Regarding the 50 mL core volume threshold used for patient selection in most late window clinical trials, our model correctly estimated infarcts larger than 50 mL with 97% (29/30) accuracy, compared to the three experts whose accuracy varied from 23% (7/30) to 47% (14/30; *p* < 0.0001). The experts failed to detect 7% (2/30) to 23% (7/30) of these large infarcts and categorized 17% (5/30) to 47% (14/30) as being < 20 mL. Our model also detected 100% (60/60) of strokes > 20 mL, of which the experts missed 18% (11/60) to 32% (19/60); indeed, as per the Bland–Altman plot (Fig. 1c), the 95% confidence interval for mean DWI-NCCT core volume measurement was under ± 17 mL overall, across all volumes. Confusion matrices for infarct estimation accuracy confirm superior model performance versus experts for estimating > 0–20 mL, > 20–50 mL, > 50 mL volume thresholds (Fig. 1d).

Our model similarly showed excellent performance not only for detection and volume estimation of suspected MCA-territory infarcts larger than 70 mL and 100 mL (Fig. 2), as well as for each of the stratified onset-to-imaging time windows (0–3 h, 3–6 h, > 6 h, Table 2), but also for detection and volume estimation of suspected infarcts in an expanded, more heterogeneous test set of 364 total CT scans (94 stroke-negative, 270 stroke-positive) with mixed territory strokes (Tables 1 and 3); in this cohort, model sensitivity was 97% and specificity 99%, for detection of infarcts larger than the 70 mL volume threshold used for patient selection in several major, randomized controlled trials of thrombectomy treatment.

**Table 2:** Model and expert reader sensitivities and specificities of Fig. 2b, stratified by 0-3 h, 3-6 h, and > 6 h symptom (sx) onset-to-NCCT times, in the “MCA-territory only” test set (**a-c**, calculated at the model’s 0 mL-threshold for infarct detection, for the n = 76 patients for whom onset-to-NCCT time data was available). Overall onset-to-NCCT times ranged from 32 min to 22.9 h, median 3.7 h.

	**Threshold**	**0 ml**	**20 mL**	**50 mL**	**70 mL**	**100 mL**
**Sx Onset-to-NCCT 0–3 h (n = 30)**						
**Model**	**Sensitivity**	**0.90 (0.79–1.0)**	**0.88 (0.76–0.99)**	**0.93 (0.84–1.00)**	**1.00 (1.00–1.00)**	**0.83 (0.70–0.97)**
	**Specificity**	**-**	**1.00 (1.00–1.00)**	**1.00 (1.00–1.00)**	**0.95 (0.87–1.00)**	**0.96 (0.89–1.00)**
**Expert 1**	**Sensitivity**	**0.47 (0.29–0.65)**	**0.25 (0.10–0.40)**	**0.14 (0.02–0.27)**	**–**	**–**
	**Specificity**	**-**	**1.00 (1.00–1.00)**	**1.00 (1.00–1.00)**	**–**	**–**
**Expert 2**	**Sensitivity**	**0.63 (0.46–0.81)**	**0.12 (0.01–0.24)**	**0.07 (0.00–0.16)**	**–**	**–**
	**Specificity**	**-**	**1.00 (1.00–1.00)**	**1.00 (1.00–1.00)**	**–**	**–**
**Expert 3**	**Sensitivity**	**0.57 (0.39–0.74)**	**0.38 (0.20–0.55)**	**0.21 (0.07–0.36)**	**–**	**–**
	**Specificity**	**-**	**1.00 (1.00–1.00)**	**1.00 (1.00–1.00)**	**–**	**–**
**Sx Onset-to-NCCT 3–6 h (n = 37)**						
**Model**	**Sensitivity**	**1.00 (1.00–1.00)**	**0.95 (0.88–1.00)**	**1.00 (1.00–1.00)**	**1.00 (1.00–1.00)**	**1.00 (1.00–1.00)**
	**Specificity**	**-**	**0.88 (0.77–0.98)**	**0.96 (0.90–1.00)**	**1.00 (1.00–1.00)**	**1.00 (1.00–1.00)**
**Expert 1**	**Sensitivity**	**0.68 (0.52–0.83)**	**0.48 (0.32–0.64)**	**0.80 (0.67–0.93)**	**–**	**–**
	**Specificity**	**-**	**1.00 (1.00–1.00)**	**1.00 (1.00–1.00)**	**–**	**–**
**Expert 2**	**Sensitivity**	**0.68 (0.52–0.83)**	**0.52 (0.36–0.68)**	**0.40 (0.24–0.56)**	**–**	**–**
	**Specificity**	**-**	**0.94 (0.86–1.00)**	**1.00 (1.00–1.00)**	**–**	**–**
**Expert 3**	**Sensitivity**	**0.73 (0.59–0.87)**	**0.52 (0.36–0.68)**	**0.60 (0.44–0.76)**	**–**	**–**
	**Specificity**	**-**	**0.94 (0.86–1.00)**	**0.96 (0.90–1.00)**	**–**	**–**
**Sx Onset-to-NCCT > 6 h (n = 9)**						
**Model**	**Sensitivity**	**1.00 (1.00–1.00)**	**–**	**1.00 (1.00–1.00)**	**1.00 (1.00–1.00)**	**1.00 (1.00–1.00)**
	**Specificity**	**-**	**1.00 (1.00–1.00)**	**1.00 (1.00–1.00)**	**1.00 (1.00–1.00)**	**1.00 (1.00–1.00)**
**Expert 1**	**Sensitivity**	**0.67 (0.36–0.97)**	**0.50 (0.17–0.83)**	**1.00 (1.00–1.00)**	**–**	**–**
	**Specificity**	**-**	**1.00 (1.00–1.00)**	**1.00 (1.00–1.00)**	**–**	**–**
**Expert 2**	**Sensitivity**	**0.67 (0.36–0.97)**	**0.75 (0.47–1.00)**	**1.00 (1.00–1.00)**	**–**	**–**
	**Specificity**	**-**	**1.00 (1.00–1.00)**	**1.00 (1.00–1.00)**	**–**	**–**
**Expert 3**	**Sensitivity**	**0.67 (0.36–0.97)**	**0.50 (0.17–0.83)**	**1.00 (1.00–1.00)**	**–**	**–**
	**Specificity**	**-**	**1.00 (1.00–1.00)**	**1.00 (1.00–1.00)**	**–**	**–**

**Table 3:** Sensitivity, specificity, AUROC, and Dice values for the full, heterogeneous test set including both MCA and non-MCA territory infarcts (n = 364 patients, Table 1), at the 0 mL, 5 mL, 10 mL, 20 mL, 50 mL, 70 mL, and 100 mL volume thresholds for detection.

	Sensitivity	Specificity	AUROC	Dice
Threshold 0 mL	0.83 (0.78–0.87)	0.64 (0.54–0.73)	0.68 (0.60–0.76)	0.36 (0.31–0.40)
Threshold 5 mL	0.75 (0.69–0.82)	0.96 (0.93–0.99)	0.77 (0.70–0.83)	0.59 (0.54–0.64)
Threshold 10 mL	0.83 (0.76–0.89)	0.99 (0.97–1.00)	0.83 (0.77–0.90)	0.66 (0.61–0.71)
Threshold 20 mL	0.89 (0.81–0.95)	0.98 (0.97–1.00)	0.91 (0.85–0.96)	0.75 (0.71–0.79)
Threshold 50 mL	0.93 (0.84–1.00)	1.00 (0.99–1.00)	0.93 (0.84–1.00)	0.78 (0.71–0.83)
Threshold 70 mL	0.97 (0.89–1.00)	0.99 (0.98–1.00)	0.97 (0.89–1.00)	0.80 (0.73–0.84)
Threshold 100 mL	0.91 (0.76–1.00)	0.99 (0.99–1.00)	0.95 (0.84–1.00)	0.80 (0.71–0.87)

The performance of our model for safe rapid estimation of infarct core, essential to patient selection for both early and late time window stroke treatments such as EVT, compared favorably not only to that of other published AI models for NCCT acute stroke detection and delineation, but also to the performance of more complex, costly, and time-consuming “advanced” CT and MR imaging techniques such as CT perfusion imaging and MR-DWI.

These results are likely in large part attributable to our large, accurately labeled training set consisting of 3566 NCCT / ground truth diffusion MRI patient pairs of early strokes (most < 6 h post-onset), for which DWI was obtained within 3-h of admission CT for stroke-positive patients (median ≤ 50 min) and within 5-days for stroke-negative patients (median ≤ 19 h) (Fig. 3a)^14–19^. It is noteworthy that both our training/validation and test sets contained predominantly small volume strokes (median DWI infarct volume estimates < 10 mL and < 30 mL, respectively; see Table 1, *Methods*). Much of the existing work on automated detection and analysis of acute stroke focuses on three approaches: imaging features engineering, ischemic region segmentation, or biomarkers computation^14^. Although some of this literature reports high performance, few of these studies are focused on early ischemic findings and limitations include small and/or poorly annotated training datasets, as well as weaker “reference standard” ground truth (e.g., ground truth based on reader consensus or on less accurate, more highly variable modalities than MR-DWI, such as CTP)^3,4,14–19^.

## Discussion

It is noteworthy that, at the 50 & 70 ml infarct volume thresholds that are clinically relevant for thrombectomy treatment decisions in MCA stroke patients (Fig. 2b), sensitivity is 97% for the model and ranges from 23 to 47% for the expert readers, for equal specificities of 99–100%. Table 3 shows similarly good results for model performance in the full, heterogeneous test set of mixed stroke subtypes at these infarct volume thresholds. These results highlight what may be one of the more important potential use cases for our model; specifically, estimation of core volume to help select MCA occlusive stroke patients with infarcts smaller than the 50 or 70 ml thresholds suggested by the major stroke therapy randomized controlled trials (RCT’s) for thrombolytic treatment inclusion^2,4^. Indeed, the “MCA-territory-only” test set was restricted to focus specifically on model performance for this clinically relevant subset of patients who are potential candidates for catheter thrombectomy.

In one published model tested on 100 CT scans, for example (median 48-min after symptom onset, IQR 27–93 min), there was moderate correlation between algorithm-predicted NCCT and expert-contoured DWI infarct estimated volumes (r = 0.76, r^2^ = 0.58), with the Bland–Altman plot 95% confidence interval for DWI-NCCT core volume measurement ranging from −59 to 80 mL, versus -18 to 16 mL for our model (Fig. 1c)^15^. Recently, a model trained on NCCT/DWI pairs showed 0.76 accuracy for < 9 h infarct detection^16^. For a different recently published model tested on 479 early and late window acute stroke CTs, there was modest correlation between NCCT predicted volumes and both CTP derived (r = 0.44, r^2^ = 0.19) and final-infarct (r = 0.52, r^2^ = 0.27) estimated volumes^17^. Another recent model showed moderate performance in correlating automated NCCT Alberta Stroke Program Early CT Scores (i.e., “ASPECTS”, a 10-point scoring system for infarct size estimates) with measured CTP (r^2^ = 0.58) and DWI (r^2^ = 0.46) core volumes^18^. Moreover, our algorithm’s accuracy is notably superior to that of the CTP derived estimated infarct volume accuracies reported in the literature (e.g., Bland–Altman plot 95% confidence interval for mean CTP-DWI core volume measurement ranging from –59 to 55 mL^19^).

Few medical artificial intelligence (AI) models to date have significantly outperformed human experts, and better-than-human detection and delineation of clinically important findings on CT or MRI cross sectional imaging has not previously been emphasized in the literature^20,21^. In one study of a convolutional neural network (CNN) for malignant melanoma detection, compared to a group of 58 dermatologists with a broad range of experience including 30 experts, the “CNN missed fewer melanomas and misdiagnosed benign moles less often as malignant”^20^. In another AI imaging study, McKinney et al. described a system for breast cancer screening mammography that outperformed US board certified radiologists “compliant with the requirements of the Mammography Quality Standards Act”^21^. There was a 5.7% reduction in false positives and a 9.4% reduction in false negatives with this system, which outperformed all human readers with an area under the receiver operating characteristic curve (AUC-ROC) of 0.740, reflecting an 11.5% improvement over the 0.625 AUC radiologist average. The authors concluded that AI has the potential to alleviate pressures on limited radiology staffing resources, as well as to discern “patterns and associations that are often imperceptible to humans”. Indeed, Fig. 3b shows two head CT’s that were interpreted as negative for stroke by all three of our neuroradiology experts, but correctly classified by our model as positive for early infarction (one of which had a large, > 125 mL estimated infarct core).

In summary, we have developed a deep learning model that leverages the high sensitivity of DWI as ground truth to automate the detection, segmentation, and volume estimation of early ischemic changes on NCCT. Although DWI remains the operational reference standard for maximally sensitive, early infarct estimation, MRI is a limited resource, not rapidly and routinely accessible in most acute care settings, such as community hospitals and rural urgent-care facilities, where only CT is likely to be available. Indeed, our deep learning platform might be especially beneficial to stroke patients in underserved areas, without 24/7 advanced imaging capability or off-hour radiologist staffing.

It is noteworthy that, because our training set used the DWI-lesion as the ground truth “operational” reference standard, we are limited in our ability to assess the intriguing possibility that CT based estimation of infarct might, in fact, be superior to that obtained by MR-DWI. Although it would be fascinating to compare our DWI trained model to a CT trained AI model for infarct detection, unfortunately, there are insufficient patients in our dataset with appropriate ground truth based on follow-up CT imaging, in the setting of early robust reperfusion, for training and testing. Interestingly, Flumazenil-PET scanning has been used for more accurate core assessment than other “operational” measures, but it’s use is also beyond the scope of this study^12^*.* Indeed, because NCCT was obtained as the first imaging exam, prior to MRI, for all cases, any bias in the study design is likely to favor MRI as being more sensitive than NCCT for infarct detection. Given, however, that the 50 mL, 70 mL, and 100 mL core volume thresholds relevant to patient selection for thrombectomy^3^ are typically estimated clinically from CT perfusion and/or high signal-to-noise ratio MR DWI images—rather than from NCCT images that have relatively poor signal-to-noise ratio and are therefore challenging to segment accurately—the ability of our AI model to accurately distinguish between different volume thresholds, based only on NCCT exams, seems especially relevant to clinical workflow.

Potential limitations of our study include focusing the testing set on patients who were potential candidates for thrombectomy. Specifically, our study focused primarily on MCA territory strokes, which limits the generalizability of our model to assess head CT scans with more varied stroke locations, mechanisms (e.g., large vessel versus non-large vessel occlusive strokes), and onset-to-imaging times, as well as the ability to assess exams performed on different CT models from different manufacturers, acquired using more varied scanning parameters (see Table 1). Moreover, in our selection of cases for both training and testing, it is unfeasible to determine what proportion of non-MCA strokes were not included.

In conclusion, the accuracy of our AI model for non-contrast head CT early stroke *detection* and *volume estimation* in a thrombectomy eligible cohort (greater-than versus less-than 50 mL), exceeds that of human experts and approaches that of ground truth MR-DWI. If prospectively validated and confirmed to be generalizable across a variety of different CT-scanner platforms, manufacturers, and acquisition protocols at different institutions, this model has the potential to be a suitable alternative for more complex, costly, and time-consuming exams, especially at primary stroke referral centers that may have limited availability of advanced CT and MR imaging techniques. Further studies are currently underway to assess the generalizability of this model for the safe rapid selection of patients for early- and late-time window, highly effective stroke treatments such as endovascular thrombectomy.

## Methods

**Figure 4:**
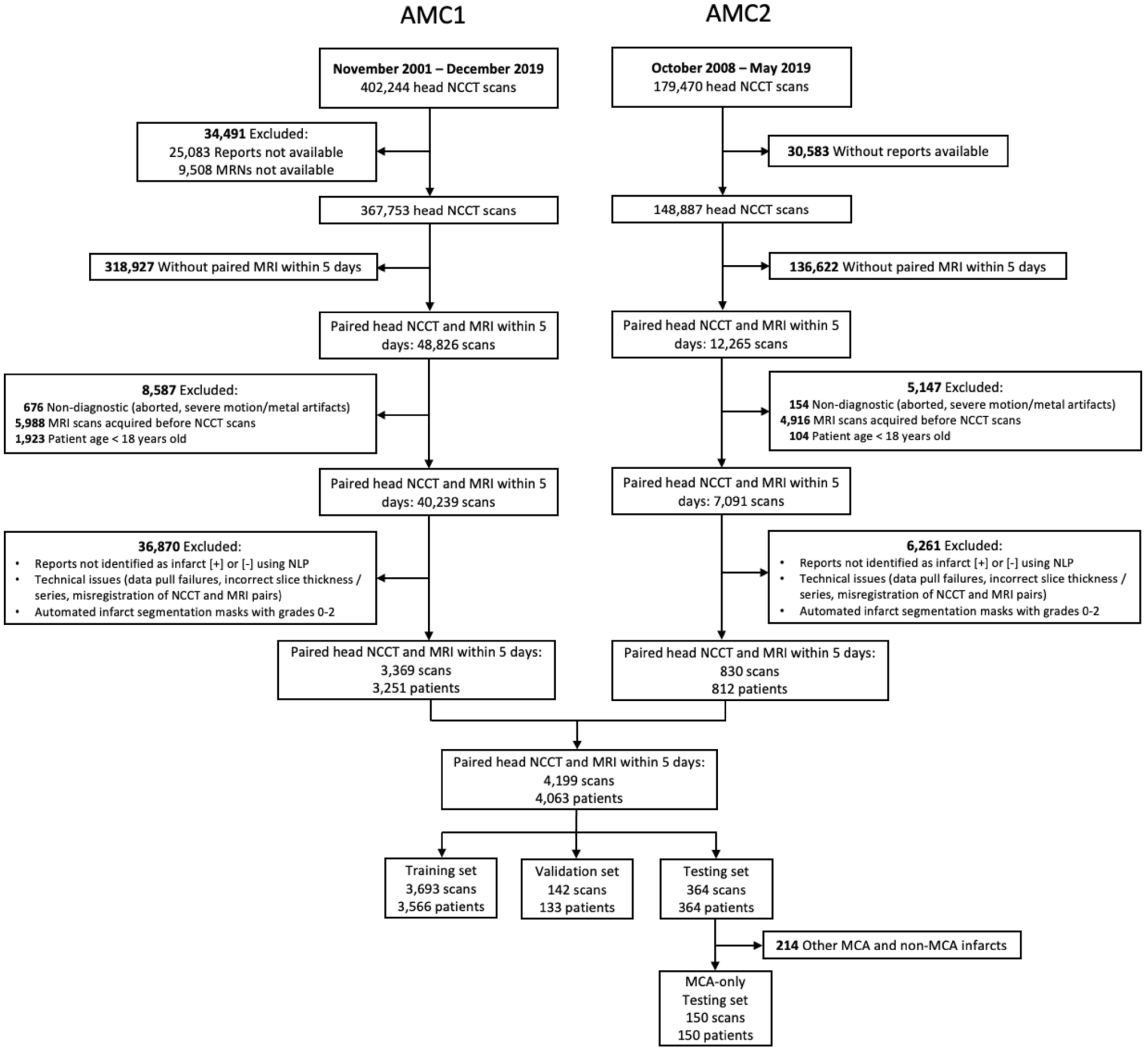
Flowchart of patient inclusion from two academic medical centers. (Legend: AMC = academic medical center, NCCT = non-contrast head CT, MRN = medical record number, NLP = natural language processing, MCA = middle cerebral artery).

This was a HIPAA-compliant retrospective study with the approval of Partners HealthCare System (now Mass General Brigham) Institutional Review Board. Inform patient consent was waived by Partners HealthCare System (now Mass General Brigham) Institutional Review Board and all methods were performed in accordance with the ethical standards of Helsinki Declaration. The dataset was identified by searching the radiology exam archive of two large US academic medical centers (AMC) for NCCT scans for which patients also had MR-DWI scans acquired within the following 5 days (AMC1 date range, 2001–2019; AMC2 date range, 2008–2019; Fig. 4). MRI reports were screened using parsing methods (keyword and sentence matching) to identify studies positive and negative for acute stroke. CNN input of stroke negative versus positive cases was based on MRI-DWI classification, with stroke-negative cases confirmed to have DWI/ADC infarct volume = 0. All images were reviewed and verified by experienced radiologists; areas of restricted diffusion in stroke-positive patients were manually segmented and the segmentation masks were inputted to the CNN. For stroke-negative exams, no segmentation masks were created.

The time difference between the NCCT and MR-DWI scans was limited to under 3 h for stroke-positive scans, to capture infarct-related physiological changes on NCCT as close as possible to the MRI ground truth, and to under 5-days for stroke-negative scans, as restricted diffusion persists for several weeks following acute stroke. NCCT was obtained prior to MR-DWI for all exam pairs included in this study. DWI was routinely performed immediately following CT for all stroke patients imaged at one of our two medical centers, as part of our standardized “stroke code” protocol, for the date ranges searched. All reports were manually reviewed by a trained radiologist. Scans were de-identified using the Radiological Society of North America Clinical Trial Processor with customized scripts.

Brain MR-DWI and Apparent Diffusion Coefficient (ADC) sequences were considered ground truth for the presence or absence of suspected acute infarction; axial DWI “b = 1000” and ADC series with slice thickness ≥ 5 mm were selected using a brain MRI series selection algorithm^23^. All images were reviewed by a trained radiologist to ensure correct classification. Infarct segmentation was performed using established methodology, including a previously developed algorithm for mask generation^24^. The automated masks were reviewed by a trained radiologist along with the corresponding MR-DWI/ADC series and radiology reports. Only segmentations with high quality were used for model development; the others were discarded or manually segmented by a trained radiologist (Osirix MD v11.0.3).

The segmented DWI stroke-positive and negative scans were paired with the corresponding NCCT scans, obtained post-symptom onset but prior to DWI acquisition. Axial CT images with slice thickness ≤ 5 mm and standard or soft kernel reconstructions, computed using routine iterative reconstruction or filtered back projection algorithms, were manually selected for model input (JKC, DC, JP, AP, JC, ES); in some cases, this resulted in several CT scans per patient (Table 1, 2nd row). Scans were excluded if they were non-diagnostic (e.g., severe metal or motion artifact). NCCT/DWI image pairs were spatially registered using the SimpleITK Python package (v1.2) with a multiscale affine transformation and mutual information loss. Registration results were assessed visually; failed or imprecisely registered images were excluded.

The resulting dataset was randomly sampled to create the training (80%), validation (10%), and testing (10%) sets (Table 1 and Fig. 4). For the purposes of this study, the model was applied to two test sets; the first, an “MCA-territory-only” subset that was restricted to 150 patients, and the second, an expanded test set of 364 patients that included these 150 plus additional patients with more varied infarct locations (Table 1). For the training and validation sets, all selected CT scans were retained for model training and validation, even if there were multiple scans per patient, in order to maximize algorithm robustness at training and enhance algorithm evaluation at validation. For the test sets, only a single CT scan per patient was used; if more than one was available, the earliest (i.e., closest to admission) within the defined post-symptom onset timeframe was used, with 5 mm-thick standard kernel reconstructed slices prioritized. The “MCA-territory-only” test set included 60 stroke-negative control patients and 90 stroke-positive patients, distributed evenly with 30 NCCT/DWI pairs in each of the > 0–20 mL, > 20–50 mL, > 50 mL estimated infarct volume categories. Only patients with strokes in the treatment-relevant middle cerebral artery vascular territory of the brain were selected for inclusion. The second test set, with more varied infarct locations, included 94 stroke-negative patients, 270 stroke-positive patients.

We developed a neural network that takes a 3D CT axial image stacks of varying number of slices as an input and outputs both a classification result and a segmentation mask. Pre-processing steps on the input 3D image are: (1) the NCCT axial slices are resampled to 5 mm thickness, and each slice are then resized to 256 × 256, with a maximum of 35 axial slices; (2) pixel intensities are clipped to window-width and center-level display range settings of 90 and 40 Hounsfield units, respectively (typical display parameters used clinically by neuroradiologists for stroke CT image interpretation^8^); and (3) the resampled image pixel values are mapped between 0 and 1. The binary masks, superimposed in the preprocessing step onto the original NCCT input slices, allows infarct volume estimation into ≤ 20 mL, > 20–50 mL, or > 50 mL categories. The input to the CNN is a 3D volume; these categories were used only for testing purposes, but not for model training. The Dice score and the classification scores were used for optimizing the model.

The network design extends the U-Net approach for biomedical image segmentation^22^. The 3D architecture is slightly modified, with an additional classification output that adds a classification component to the loss function, while maintaining segmentation and classification output consistency. Adding a classification component improved performance compared to using the Dice loss alone, as several very small stroke masks in our dataset could contribute disproportionally to lower the Dice score. The model was developed using Python 3.6 and Tensorflow 1.13.1. Although the input image size is fixed in the axial in-plane dimensions, the framework can process 3D image volumes with varying numbers of slices. The architecture otherwise follows a U-Net design, with 6 down-sampling blocks (composed of 3 × 3 convolutions, batch normalization, and maximum pooling layers, followed by ReLU activation) and 6 up-sampling blocks. It differs from a classical architecture in that the pooling operations are done at the slice level only, with shape (2, 2, 1), rather than between slices. This avoids unintended interpolation effects when the slice thickness is large. The neural network is optimized using a loss function that combines a differentiable Dice loss (for segmentation) and a cross entropy loss (for study-level classification). The combination of the losses is a linear combination, using a constant that is set manually. This parameter was tuned manually by training multiple models with different values between 0 and 1.

We applied geometrical and pixel intensity-based data augmentation techniques at the 3D volume level, including a combination of in-slice rotations and translations, scaling, right-left flipping, and both Gaussian and Poisson random noise. At each epoch, each transformation (applied in the image space) was drawn with a probability of 0.5, and if applicable, the transformation parameters were randomly modified with a probability of 0.95.

To control for data imbalance in our training set, we developed a batch sampling strategy. For each batch: (1) selecting 8 stroke-positive and 4 stroke-negative scans, to ensure there is always a sufficient number of positives in a batch; and (2) selecting 7 scans acquired from General Electric (GE) CT platforms and 1 from Siemens platforms for stroke positive patients, and 2 from GE and 1 from Siemens for stroke negative patients, to reflect the manufacturer distribution of scanner platforms typically available for emergency department “stroke code” use at both institutions. Moreover, among stroke-positive scans, there was a large percentage of very small infarcts (< 1 mL) in the training set (455/1896 = 24%). Because signal-to-noise ratio, and hence CT conspicuity, of these tiny infarcts is likely to be poor—which could contribute to both decreased accuracy for stroke detection and increased error rate for small structure segmentation, impacting Dice loss—we studied the effects on model performance of excluding infarcts smaller than 1 or 5 mL in our analyses (Fig. 1a). Those results suggest that, for future clinical implementation, exclusion of infarcts smaller than 1 mL might provide an appropriate operating point on the ROC curve as a trade-off between optimizing both sensitivity and specificity for stroke *detection*.

Our neural network was trained using the Adam optimizer; network parameters were initialized with the uniform approach proposed by Glorot and Bengio^25^. The learning rate was reduced by a factor of 0.75 when the validation loss did not improve after 20 epochs. Our network trained for a maximum of 200 epochs, processed using NVIDIA 4 GPU Tesla V100 with 32 Gb RAM, allowing batch sizes of twelve 3D volumes; training a single model took approximately 2.5 days. Such computationally demanding training was prohibitive for extensive hyperparameter search; approximately 400 different models were trained during the roughly 2-year development cycle. Hyperparameter search was performed manually with a grid search approach; the following parameters were tuned: learning rate, loss weights, batch sampling strategy (random uniform, positive/negative sampling, manufacturer sampling), exclusion/inclusion of infarcts (< 1 mL, < 5 mL), and size of the first convolutional layer. Hyperparameter tuning was performed on the validation and training sets exclusively. Next, a small set of models were selected according to pre-defined performance metrics (Dice, ROC-AUC, sensitivity/specificity). These models were presented to a panel of several experienced radiologists, blinded to the specific model parameters, but with the performance metrics and a random, representative sample of segmentation results available for review for each model. The experts ranked these models and provided justification for their ratings; majority voting was used to select the final model to use for MCA-territory-only test set comparison to three, independent, expert neuroradiologists (Fig. 1a).

For model metrics, 95% confidence intervals were computed using either the simple asymptotic method (for classification metrics) or bootstrapping technique (for continuous values, bootstrap size 500). Bland–Altman plot analysis was performed with MedCalc software. Python (v3.7) with NumPy package (v1.2) was used for all other statistical calculations, including but not limited to ROC curve analyses and linear regression. A *p* < 0.05 level of confidence was considered statistically significant.

## Data Availability

The training, validation, and test datasets generated for this study are protected patient information. Some data may be available for research purposes from the corresponding author upon reasonable request.

## Code availability

The code base for the deep-learning framework makes use of proprietary components and we are unable to publicly release the full code base. However, all experiments and implementation details are described in sufficient detail in the Methods to enable independent replication with non-proprietary libraries.

## References

[1] Lindsay MP (2019). World stroke organization (WSO): Global stroke fact sheet 2019. Int. J. Stroke.

[2] Nogueira RG, *et al*., DAWN Trial Investigators. Thrombectomy 6 to 24 Hours after Stroke with a Mismatch between Deficit and Infarct. *N. Engl. J. Med*. **378**, 11–21 (2018). 10.1056/NEJMoa1706442 29129157

[3] Leslie-Mazwi TM (2016). Endovascular stroke treatment outcomes after patient selection based on magnetic resonance imaging and clinical criteria. JAMA Neurol..

[4] Campbell BCV, *et al*., HERMES collaborators. Penumbral imaging and functional outcome in patients with anterior circulation ischaemic stroke treated with endovascular thrombectomy versus medical therapy: a meta-analysis of individual patient-level data. *Lancet Neurol*. **18**, 46–55 (2019). 10.1016/S1474-4422(18)30314-4 30413385

[5] Nogueira RG, *et al.*, Trevo Registry and DAWN Trial Investigators. stroke imaging selection modality and endovascular therapy outcomes in the early and extended time windows. *Stroke*. **52**, 491–497 (2021). 10.1161/STROKEAHA.120.031685 33430634

[6] Kim BJ (2021). endovascular treatment after stroke due to large vessel occlusion for patients presenting very late from time last known well. JAMA Neurol..

[7] Berkhemer OA (2015). A randomized trial of intraarterial treatment for acute ischemic stroke. N. Engl. J. Med..

[8] Lev MH (1999). Acute stroke: improved nonenhanced CT detection–benefits of soft-copy interpretation by using variable window width and center level settings. Radiology.

[9] Camargo EC (2007). Acute brain infarct: detection and delineation with CT angiographic source images versus nonenhanced CT scans. Radiology.

[10] Mullins, *et al*. CT and Conventional and Diffusion-Weighted MR Imaging in Acute Stroke: Study in 691 Patients at Presentation to the Emergency Department. *Radiology* **224**, 353–60 (2002). 10.1148/radiol.2242010873 12147827

[11] Fiebach JB (2002). CT and diffusion-weighted MR imaging in randomized order: Diffusion-weighted imaging results in higher accuracy and lower interrater variability in the diagnosis of hyperacute ischemic stroke. Stroke.

[12] Heiss WD (2004). Probability of cortical infarction predicted by flumazenil binding and diffusion-weighted imaging signal intensity: a comparative positron emission tomography/magnetic resonance imaging study in early ischemic stroke. Stroke.

[13] Sims JR (2009). ABC/2 for rapid clinical estimate of infarct, perfusion, and mismatch volumes. Neurology.

[14] Mikhail P, Le MGD, Mair G (2020). Computational image analysis of nonenhanced computed tomography for acute ischaemic stroke: A systematic review. J. Stroke Cerebrovasc. Dis..

[15] Qiu W (2020). Machine learning for detecting early infarction in acute stroke with non-contrast-enhanced CT. Radiology.

[16] Pan J (2021). Detecting the early infarct core on non-contrast CT images with a deep learning residual network. J. Stroke Cerebrovasc. Dis..

[17] Bouslama M (2021). Noncontrast computed tomography e-stroke infarct volume is similar to RAPID computed tomography perfusion in estimating postreperfusion infarct volumes. Stroke.

[18] Nagel S (2020). e-ASPECTS derived acute ischemic volumes on non-contrast-enhanced computed tomography images. Int. J. Stroke..

[19] Schaefer PW (2015). Limited reliability of computed tomographic perfusion acute infarct volume measurements compared with diffusion-weighted imaging in anterior circulation stroke. Stroke.

[20] Haenssle HA (2018). Man against machine: Diagnostic performance of a deep learning convolutional neural network for dermoscopic melanoma recognition in comparison to 58 dermatologists. Ann. Oncol..

[21] McKinney SM (2020). International evaluation of an AI system for breast cancer screening. Nature.

[22] Ronneberger O, Fischer P, and Brox T. U-Net: Convolutional Networks for Biomedical Image Segmentation. Preprint at https://arxiv.org/abs/1505.04597v1 (2015).

[23] Gauriau R (2020). Using DICOM metadata for radiological image series categorization: A feasibility study on large clinical brain MRI datasets. J Digit. Imaging..

[24] Pedemonte S (2018). Detection and delineation of acute cerebral infarct on DWI using weakly supervised machine learning. Med. Image Comput. Comput. Assist. Interv. (MICCAI).

[25] Glorot X, Bengio Y (2010). Understanding the difficulty of training deep feedforward neural networks. Proc. Thirteenth Int. Conf. Artif. Intell. Stat..

